# Phosphorus-drought interaction modulates growth dynamics and essential oil biosynthesis in *Rosmarinus officinalis*

**DOI:** 10.3389/fpls.2025.1646658

**Published:** 2025-10-30

**Authors:** Mohamed Alfalah, Rachid Bouharroud, Adnane Beniaich, Fatima El Aroussi, Mohamed El Gharous, Karim Lyamlouli

**Affiliations:** ^1^ College of Sustainable Agriculture and Environmental Sciences, Agricultural Innovation and Technology Transfer Center (AITTC), Mohammed VI Polytechnic University (UM6P), Ben Guerir, Morocco; ^2^ Integrated Crop Production Research Unit, Regional Center of Agricultural Research of Agadir, National Institute of Agricultural Research, Rabat, Morocco; ^3^ Faculty of Science and Technology, University Cadi Ayyad, Marrakech, Morocco; ^4^ College of Sustainable Agriculture and Environmental Sciences, AgroBioSciences Department (AgBs), Mohammed VI Polytechnic University (UM6P), Ben Guerir, Morocco

**Keywords:** rosemary, *R. officinalis*, drought, phosphorus, essential oil

## Abstract

This study aimed to investigate the combined effects of drought stress and phosphorus fertilization on the morphological, physiological, and biochemical traits of R. *officinalis*, with a focus on essential oil yield and composition. Rosemary plants were subjected to three irrigation levels (I1=80%FC; I2=50%; I3: 30% of soil moisture content at field capacity), and three phosphorus levels (P10=10 kg/ha; P25=25 kg/ha; P50= 50 kg/ha). Increasing drought stress significantly reduced growth indicators such as leaf area index (LAI), stem length, stem diameter, root and shoot biomass, and root traits, while moderate drought induced root elongation, indicating adaptive responses. Phosphorus significantly influenced stem diameter and LAI. Drought stress altered stomatal conductance and chlorophyll content, but these were maintained under moderate phosphorus supply and moderate water stress (I2P25). This treatment also resulted in the highest essential oil yield, up to 113%, compared to well-watered controls. Furthermore, I2P25 markedly enhanced the accumulation of key oxygenated monoterpenes, with relevant increases in endo-borneol, l-α-terpineol, verbenone, camphor, terpineol, and linalool. In contrast, severe drought shifted the oil profile toward monoterpene hydrocarbons. Principal Component Analysis confirmed distinct metabolic clustering at I2P25, indicating an optimal balance between stress and nutrient availability. These findings suggest that moderate drought coupled with optimal phosphorus supply improves essential oil quality and yield without compromising biomass, offering a sustainable cultivation strategy for rosemary in water-limited environments. Principal Component Analysis (PCA) supported this result, revealing distinct grouping under moderate drought (I2) and optimal phosphorus doses (P25), consistent with a metabolic tipping point marked by enhanced biosynthesis of oxygenated monoterpenes. These findings suggest that moderate drought, in combination with optimal phosphorus input, can improve essential oil quality and yield without severely compromising biomass, providing a strategic cultivation approach for rosemary under water-limited environments.

## 1 Introduction

For centuries, aromatic and medicinal plants (AMPs) have been used in medicine, perfumery, and culinary traditions worldwide. These plants contain valuable bioactive compounds with therapeutic potential, making them economically and ecologically significant (Amarti et al., 2011). Among them, rosemary (*R. officinalis* L.), a member of the Lamiaceae family, is widely cultivated for its essential oil, which exhibits antimicrobial, antioxidant, and anti-inflammatory properties (Oualdi et al., 2021; Azizi et al., 2022). The essential oil of rosemary is rich in monoterpenes such as α-pinene, camphor, and 1,8-cineole, which are highly valued in the pharmaceutical, cosmetic, and food industries (Shahina et al., 2022). The increasing global demand for rosemary essential oil underscores the importance of optimizing crop management strategies, particularly under challenging environmental conditions. In semi-arid and arid regions, the successful cultivation of rosemary is frequently hindered by abiotic stresses such as drought and nutrient limitations, which can significantly impair plant growth and essential oil yield (Nogués et al., 2015). Abiotic stress, particularly drought, has direct effects on rosemary growth, morphology, and essential oil biosynthesis. Water deficits impair photosynthesis, nutrient uptake, and secondary metabolite production, ultimately affecting essential oil yield and quality (Alarcón et al., 2006; Ormeño et al., 2007). Under drought stress, plants undergo physiological and biochemical changes, such as stomatal closure, reduced chlorophyll content, and altered metabolic pathways, to cope with water scarcity (Chaves et al., 2011). Interestingly, controlled drought stress has been shown to enhance certain secondary metabolites, suggesting potential benefits when carefully managed (Selmar and Kleinwächter, 2013). For example, moderate drought stress can increase the concentration of monoterpenes, such as verbenone and linalool, which are key components of rosemary essential oil (Kulak, 2020).

Alongside water availability, phosphorus (P) is a key macronutrient essential for plant growth, influencing photosynthesis, energy transfer, and metabolic adjustments (Kayoumu et al., 2023; Santoro et al., 2024). However, phosphorus uptake is often diminished under drought stress, as water availability is the primary driver of phosphorus transport to the roots through diffusion. In addition, water scarcity limits root activity, nutrient solubility, and translocation (Khan et al., 2023). Moreover, phosphorus from fertilizers is highly susceptible to being fixed in most soils, leading to inefficient absorption by plants (López-Arredondo et al., 2014). Phosphorus absorption by plants is mainly by diffusion and interception. Therefore, optimizing phosphorus application under drought conditions is crucial for improving P use efficiency and minimizing environmental impacts.

Researchers have conducted significant research on the independent effects of drought on essential oil production, but they have not sufficiently explored the combined effects of drought stress and P fertilization in rosemary specifically. Although several studies in medicinal and non-medicinal plants (e.g., chamomile, soybean) have reported that phosphorus can modulate drought response by enhancing growth and essential oil yield (Jeshni et al., 2017; Chen et al., 2025), these effects remain underexplored in rosemary, which differs physiologically and biochemically from other Lamiaceae species. Some studies suggest that phosphorus supplementation can help counteract the adverse effects of drought on plant growth by enhancing root architecture, optimizing photosynthetic activity, and strengthening antioxidant defense mechanisms (Bechtaoui et al., 2021). However, other studies indicate that excessive phosphorus under severe drought can exacerbate stress-induced reductions in photosynthesis and biomass production (Vukmirović et al., 2024). Given the conflicting findings on the role of phosphorus under drought stress, it is essential to elucidate the physiological and biochemical mechanisms through which phosphorus interacts with drought stress in rosemary.

The goal of this study is to investigate the combined effects of drought stress and phosphorus fertilization on rosemary’s growth, physiological performance, and essential oil yield and composition. While both drought and phosphorus availability are known to independently affect plant physiology, there is a limited literature addressing their interactive effect in *R.officinalis* particularly in relation to essential oil biosynthesis and composition. In this context, we hypothesize that controlled P fertilization could plausibly mitigate some adverse effects of drought stress on the physiological performance of rosemary, balancing the composition and quality of the essential oils and increasing the concentration of bioactive monoterpenes. This research assesses how different drought intensities influence plant growth and metabolism, explores the potential of P to mitigate drought responses, and examines its role in modulating essential oil composition. By addressing this knowledge gap, the study seeks to offer agronomic insights into optimizing phosphorus fertilization under drought conditions, thereby contributing to sustainable rosemary production and enhanced essential oil yield in semi-arid environments.

## 2 Materials and methods

### 2.1 Plant material

This study was conducted in a semi-controlled greenhouse at the Agricultural Innovation and Technology Transfer Center (AITTC), of Mohammed VI Polytechnic University (UM6P) (Benguerir, Morocco; 32°13’12.0”N, 7°53’33.5”) during the period from February to May 2024. The plant material consisted of nine-month-old rosemary (*R. officinalis*) plants, sourced from a nursery in Aghmat, Marrakech. and the plants were transplanted into pots (3L) filled with a substrate (1/3 sand and 2/3 perlite, v/v). The sand was sieved, washed with distilled water, and air-dried before mixing with perlite. The substrate composition was selected to eliminate the effects of soil heterogeneity and ensure consistent nutrient and moisture availability across treatments.

### 2.2 Experimental design

A split-plot experimental design was implemented to evaluate the combined effects of drought stress and phosphorus fertilization. The main plot consisted of three irrigation levels:

- Well-watered (80% field capacity, FC) - I1
- Moderate drought stress (50% FC) - I2
- Severe drought stress (30% FC) - I3

The P-fertilization was calculated based on Singh and Guleria (2013).

The subplots consisted of three phosphorus fertilization levels:

- Low (10 kg/ha P): P10
- Optimal (25 kg/ha P): P25
- High (50 kg/ha P): P50

This resulted in nine treatment combinations, each replicated five times, totaling 45 experimental units. Phosphorus fertilizer was provided as a single dose of Triple Superphosphate (TSP) immediately after transplantation. To maintain consistent nutrient conditions, a modified Hoagland solution was applied weekly, excluding phosphorus, to separate its effects on plant metabolism (Wiser and Blom, 2016).

### 2.3 Irrigation and field capacity determination

Irrigation was applied three times a week to maintain soil moisture at the target field capacity (FC) throughout the experiment. FC was determined by measuring the weight difference of a substrate sample at saturation and dryness statuses using the following method described by Beniaich et al. (2023), using the Equation 1:


(1) $$FC=\frac{Weight\;of\;wet\;substrate-Weight\;of\;dry\;substrate}{Weight\;of\;dry\;substrate}\times 100$$

Soil moisture was evaluated using an ML3 ThetaProbe (Delta-T Devices, UK) to ensure precise water application across treatments (Delta-T Devices, 2017).

### 2.4 Growth and morphological parameters

Plant growth was monitored monthly by measuring stem length and stem collar diameter. Stem parameters were recorded using a digital caliper. The leaf area index (LAI) was measured at midday using a SunScan Canopy Analysis System (Delta-T Devices, UK). At the end of the experiment, biomass partitioning was conducted by separating aerial and root portions. Shoot biomass was dried at 40°C to preserve essential oil integrity, while root biomass was dried at 60°C until a constant weight was reached after 3 days. Roots were carefully spread in a plastic tray and scanned using an Epson Perfection LA2400 scanner. Root morphological variables were determined using the WinRHIZO system (Regent Instruments, Quebec, Canada), following the method described by Chtouki et al. (2021).

### 2.5 Photosynthetic pigment, stomatal conductance and P uptake parameters

Physiological performance under drought and phosphorus stress was assessed by measuring chlorophyll content and stomatal conductance. Chlorophyll content was assessed once a month using an SPAD-502Plus chlorophyll meter (Konica Minolta, Japan). Stomatal conductance was measured on fully expanded mature leaves using an AP4 leaf porometer (Delta-T Devices Ltd, UK), following the method proposed by Ezzine et al. (2023).

Chlorophyll *a*, chlorophyll *b*, and carotenoid contents were measured using the method described by Er-rqaibi et al. (2025). Briefly, 2 ml of 80% (v/v) acetone was mixed with 8 mg of finely ground leaf biomass. Then the mixture was centrifuged for 30 minutes at room temperature at 12,000 g. Carefully, the supernatant was extracted for examination. Using a microplate reader (BMG Labtech, Fluostar Omega, Germany), the absorbance of the recovered supernatant was measured at three distinct wavelengths: 662 nm, 642 nm, and 470 nm. The following formulas were used to determine the levels of total carotenoids, chlorophyll *a*, and chlorophyll *b* (Equations 2–4).


(2) $$Chlorophyll\;a\;(\text{µ}g\cdot mL^{-1})=12.5\;\times A_{662}-2.79\times A_{642}\;$$

(3) $$Chlorophyll\;b\;(\text{µ}g\cdot mL^{-1})=21.5\times A_{642}-5.10\times A_{662}\;$$

(4) $$Total\;Carotenoids\;(\text{µ}g\cdot mL^{-1})=\frac{1000\times A_{470}-1.82ch\;(a)-85.02ch\;(b)}{198}$$

Phosphorus uptake was calculated using the following formula described by Chowdhury et al., 2021 (Equation 5):


(5) $$\begin{array}{c} \text{Phosphorus Uptake}\\ =\text{Biomass }(\text{dry weight})\times \text{Phosphorus Concentration} \end{array}$$

### 2.6 Gas chromatography-mass spectrometry analysis of essential oil

The EO percentage was determined using the W/W method and the following formula (Equation 6) (Khademi Doozakhdarreh et al., 2022):


(6) $$EO\%=\frac{\;EO\;weight\;(g)}{plant\;dry\;weight\;(g)}\times 100$$

Essential oils were extracted from 10 g of dried leaves per sample using Clevenger-type hydrodistillation for three hours. The collected oils were stored at 4°C until chemical analysis. The composition of essential oils was determined using Gas Chromatography-Mass Spectrometry (GC-MS) on a (GCMS-TQ8040 SHIMADZU, JAPAN. Rtx-5MS fused-bond column (30 m length, 0.25 mm internal diameter, and 0.25 μm film thickness, Restek, PA, USA). The temperature program for analysis was set as follows: The initial temperature was 50°C, increasing at a rate of 5.5°C/min to 300°C, with a final hold of three minutes. The injection mode was set to split at 250°C, using helium as the carrier gas at a flow rate of 1.5 mL/min. The detection system operated at 200°C (ion source temperature) and 280°C (interface temperature), covering a mass range of 50–500 m/z. Compound identification was performed by comparing spectra with the NIST 2017 spectral database (Naboulsi et al., 2023).

### 2.7 Statistical analysis

All statistical analyses were conducted using R software (version 2024.04.2, R Foundation for Statistical Computing, Vienna, Austria). Data normality was assessed using the Shapiro-Wilk test, and a two-way ANOVA was performed to evaluate the effects of irrigation and phosphorus fertilization on growth, physiological traits, and essential oil composition. *Post-hoc* comparisons were conducted using Tukey’s Honest Significant Difference (HSD) test at p < 0.05. Data visualization was performed using the ggplot2 package in R, with error bars representing mean ± standard error (SE). Principal component analysis (PCA) was performed to understand the relationships between evaluated variables, including morphological and physiological variables, shoot and root biomass, and essential Oil composition, using the R package “factoextra”.

## 3 Results

### 3.1 Morphological traits

#### 3.1.1 Leaf area index

**Figure 1:**
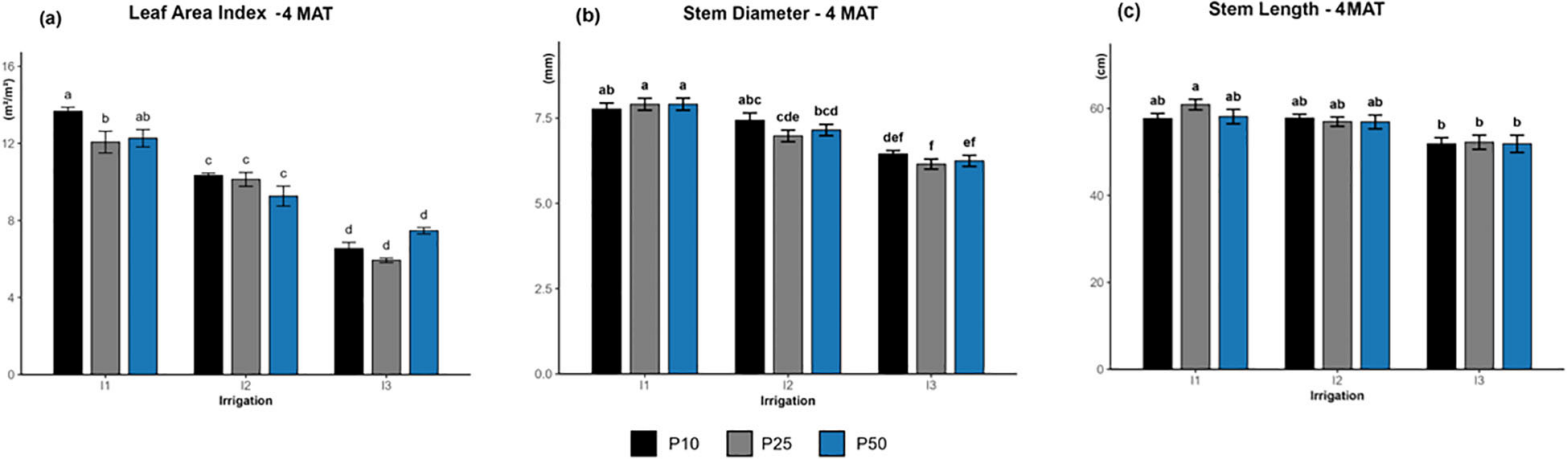
Effect of different levels of drought stress and phosphorus doses on leaf area index **(a)**, stem diameter **(b)** and stem length **(c)** of *R. officinalis* at four months after transplantation (MAT), bars represent mean values ± standard deviation. Bars sharing the same letter are not significantly different according to Tukey’s HSD test (P < 0.05).

The two-way ANOVA revealed a significant interaction between irrigation levels and phosphorus rates (p = 0.00041; **Figure 1a**). LAI values were highest under well-watered conditions (I1), especially I1P10 (13.67 m²/m²), followed by I1P50 (12.27 m²/m²) and I1P25 (12.07 m²/m²). The medium values were recorded under moderate irrigation (I2) across all phosphorus levels, while severe drought (I3) showed the lowest LAI values at I3P25 (5.93 m²/m²).

#### 3.1.2 Stem diameter and stem length

Stem diameter increased under higher irrigation levels (**Figure 1b**), with the largest diameters observed in I1 treatments (7–8 mm) and the smallest under I3 treatments (approximately 5–6 mm). Irrigation significantly affected all growth stages from the 1^st^ to the 4^th^ month after Transplantation (MAT) (*p* < 0.05), with the effect becoming particularly pronounced at the 4^th^ MAT (*p* < 0.0001). In contrast, phosphorus levels had a significant influence on the 1^st^ MAT and 3^rd^ MAT (*p* < 0.05) but showed no significant effect at the 2^nd^ MAT and 4^th^ MAT (*p* > 0.05). Similarly, stem length increased consistently under higher irrigation levels (**Figure 1c**). At the 4^th^ MAT, the longest stems were recorded under I1 treatments (63–69 cm), followed by I2 treatments (55–61 cm), and finally the shortest lengths were identified in I3 treatments (50–56 cm). This pattern highlights the positive impact of adequate water supply on stem growth. The significant effect of irrigation on stem length was observed only at later growth stages, with significance emerging at 3 MAT (*p<0.01*) and becoming highly significant at 4 MAT *(p<0.0001)*. Conversely, phosphorus did not significantly affect stem length at all growth stage (*p>0.05*), and no significant interaction between irrigation and phosphorus levels was detected across all stages (p>0.39). Tukey’s test at 4^th^ MAT further illustrated that I1P25 formed the superior treatment group, while all I3 treatments clustered in the lowest one, highlighting the limiting impact of drought stress on shoot growth at this stage.

#### 3.1.3 Shoot and root dry biomass

**Figure 2:**
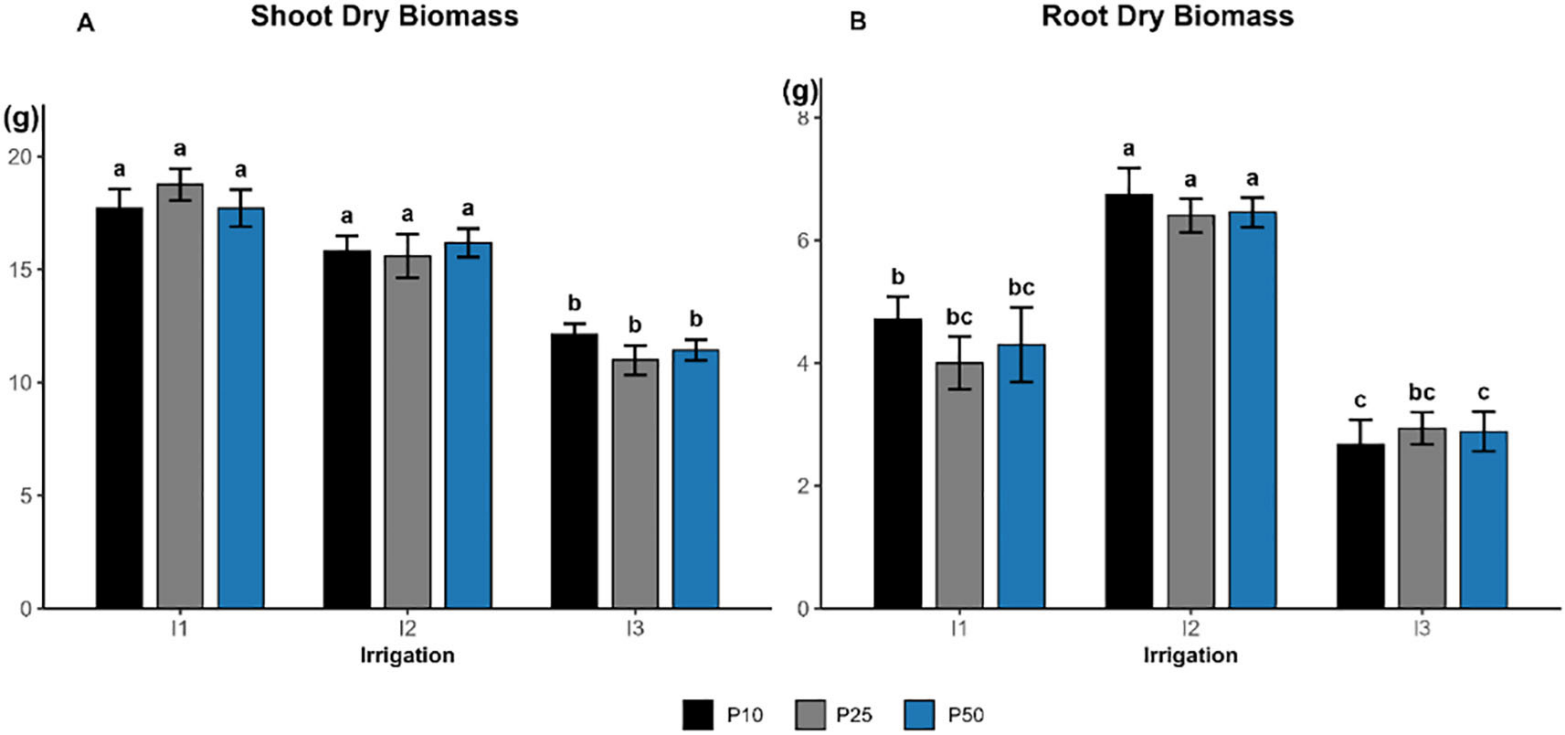
Effect of different levels of drought stress and phosphorus doses on the shoot **(A)** and root dry biomass **(B)** of *R. officinalis*, bars represent mean values ± standard deviation. Bars sharing the same letter are not significantly different according to Tukey’s HSD test (P < 0.05).

Shoot dry biomass was significantly influenced by irrigation (*p < 0.0001*; **Figure 2A**), whereas phosphorus (*p = 0.987*) and the interaction (*p = 0.503*) had no significant effect. The highest shoot biomass was observed under well-watered (I1) treatment across all phosphorus doses, attaining 18.76 g, 16.85 g, and 17.11 g, respectively, as well as under all moderate irrigation. The lowest shoot biomass values were associated with severe drought conditions across all phosphorus treatments, ranging between 11 and 12.13 g., indicating a shift in response to reduced water availability.

**Figure 3:**
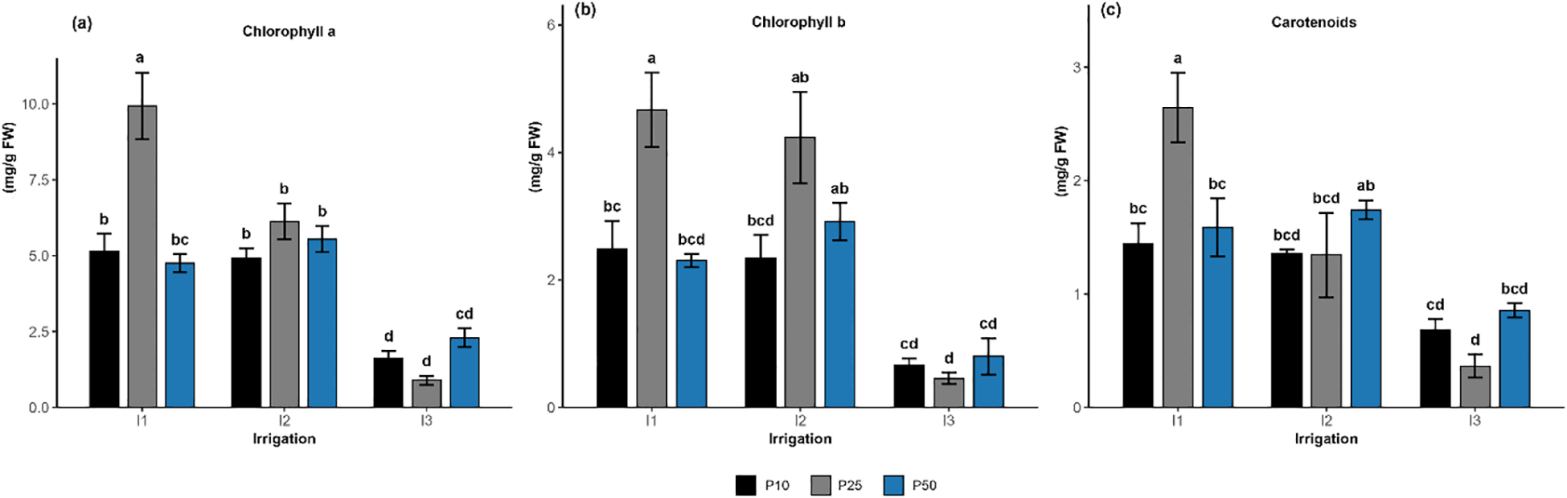
Effect of different levels of drought stress and phosphorus doses on chlorophyll *a* **(a)**, chlorophyll b **(b)**, and carotenoids content **(c)** of *R. officinalis*, bars represent mean values ± standard deviation. Bars sharing the same letter are not significantly different according to Tukey’s HSD test (*P < 0.05*).

Root dry biomass was significantly affected by irrigation (*p < 0.0001*; **Figure 3B**), whereas phosphorus (p = 0.659) and the interaction (*p = 0.764*) had no significant influence. The highest values were recorded under moderate drought treatments (I2), with a peak of 6.74 g in I2P10, which all belonged to the top statistical group. In contrast, root dry biomass declined under both well-watered (I1) and severe drought (I3) treatments across all phosphorus levels, dropping below 3 g under severe drought (I3).

#### 3.1.4 Root morphological traits

**Figure 4:**
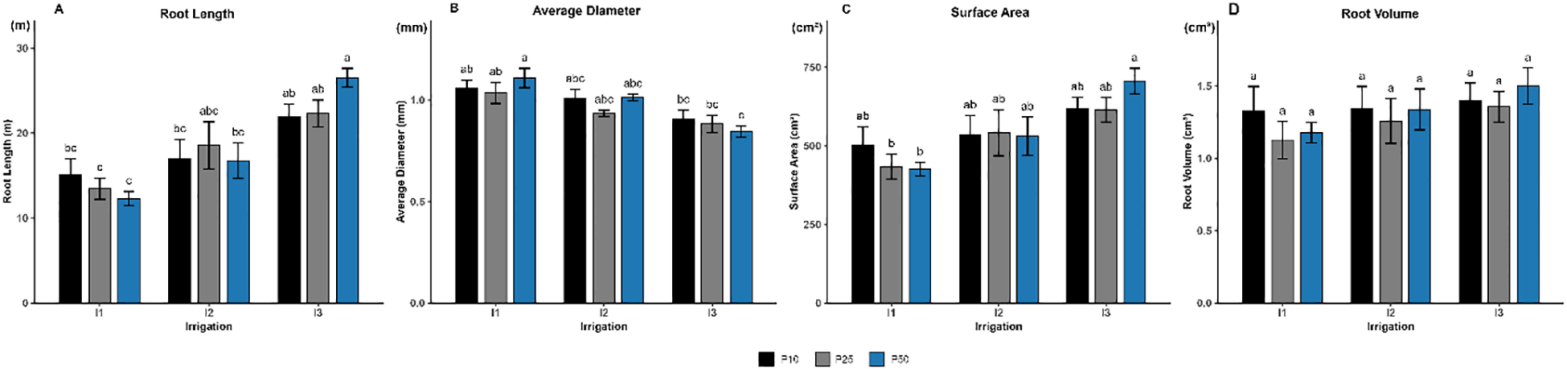
Effect of different levels of drought stress and phosphorus doses on root morphological traits of *R. officinalis*: **(A)** Root length, **(B)** Average diameter, **(C)** Surface area, and (D) Root volume. bars represent mean values ± standard deviation. Bars sharing the same letter are not significantly different according to Tukey’s HSD test (P < 0.05).

Root length was significantly influenced by irrigation levels (p < 0.001; **Figure 4A**), whereas phosphorus and the interaction had no significant effects (p > 0.05). Plants subjected to severe drought stress (I3P10, I3P25, and I3P50) exhibited the greatest root elongation, reaching 21.89 m, 22.31 m, and 26.53 m, respectively. In contrast, the shortest roots were recorded under well-watered conditions, particularly in the I1P25 (13.45m) and I1P50 (12.3 m) treatments.

In parallel, average root diameter was also significantly affected by irrigation *(p < 0.001*; **Figure 4B**), while neither phosphorus fertilization *(p = 0.368)* nor the interaction term *(p = 0.475)* had a significant effect. The thickest roots, averaging 1.11 mm, were observed under well-watered conditions (I1), demonstrating that adequate moisture promotes radial root growth. Under moderate drought (I2), root diameter exhibited a modest decrease but remained relatively stable across phosphorus levels, ranging from 0.93 to 1.01 mm. In contrast, severe drought (I3) led to a further reduction in root diameter, with the lower values shown in I3P50 averaging to 0.85 mm.

Root surface area followed a similar trend (**Figure 4C**), being also significantly influenced by irrigation levels (p < 0.001), while phosphorus and the interaction term had no significant effects (*p = 0.818* and p = 0.552, respectively). All treatments under severe drought (I3) recorded the largest root surface areas, ranging between 614 and 706 cm^2^, suggesting a compensatory root response to enhance water uptake. Treatments under moderate drought (I2) also maintained relatively high surface areas across all phosphorus levels. Conversely, well-watered plants (I1) showed the lowest root surface areas, particularly in I1P25 and I1P50, which were significantly lower and fell below 500 cm^2^.

**Figure 5:**
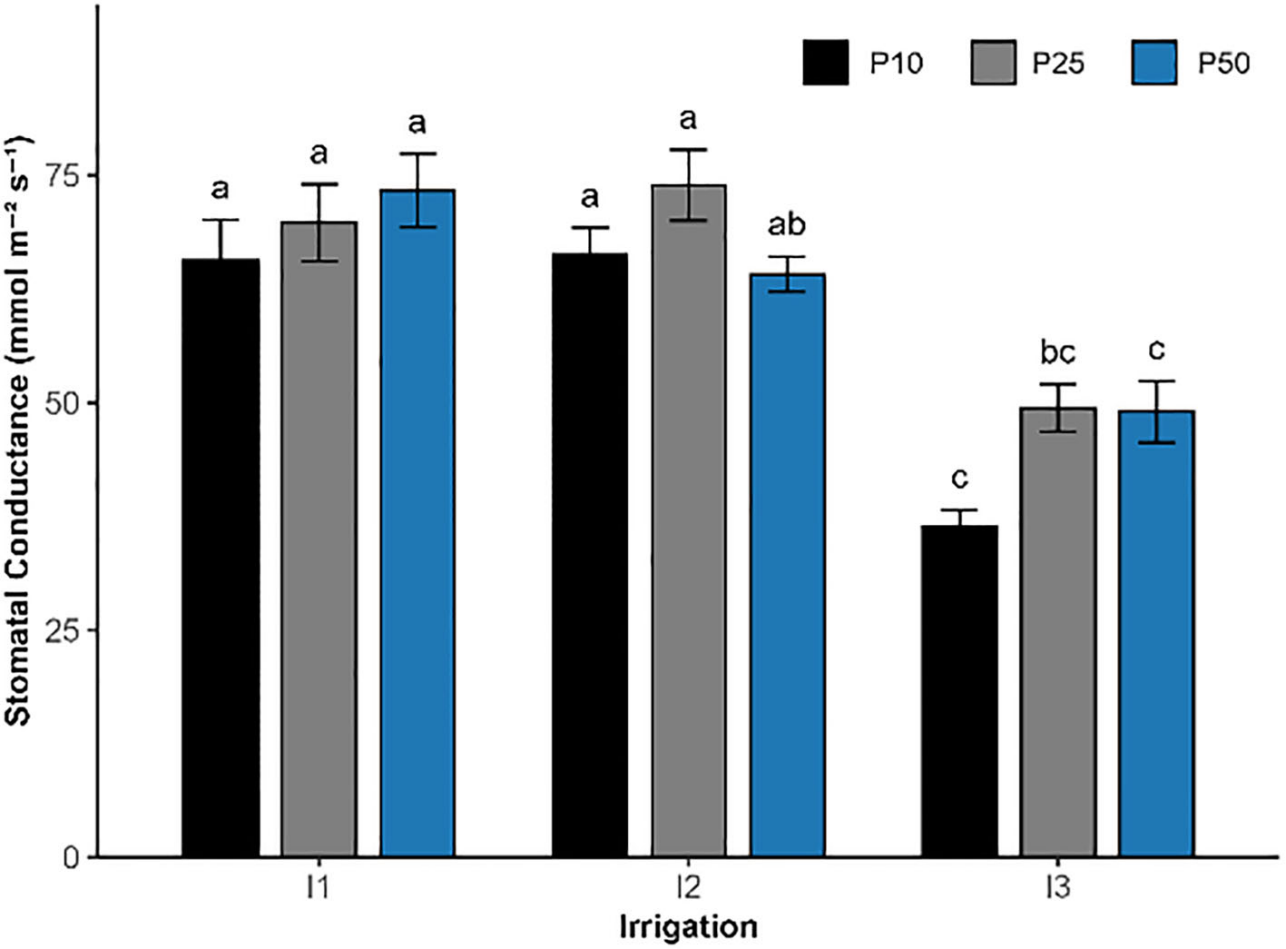
Effect of different levels of drought stress and phosphorus doses on stomatal conductance of *R. officinalis*, bars represent mean values ± standard deviation. Bars sharing the same letter are not significantly different according to Tukey’s HSD test (P < 0.05).

However, there were no significant effects of irrigation *(p = 0.174)*, nor phosphorus (p = 0.552), or their interaction (p = 0.914) on root volume of rosemary (**Figure 5D**), suggesting that this trait remained stable regardless of water or phosphorus availability.

### 3.2 Physiological traits

#### 3.2.1 Stomatal conductance

Stomatal conductance was significantly influenced by irrigation (p < 0.05) and phosphorus fertilization (p = 0.00997; **Figure 5**), while their interaction was not statistically significant (p = 0.1279). The highest conductance was observed under moderate drought (I2) combined with 25 kg/ha phosphorus (I2P25), reaching 73.93 mmol m^−2^ s^−1^, followed closely by I1P50 (73.33 mmol m^−2^ s^−1^) and I1P25 (69.80 mmol m^−2^ s^−1^). In contrast, the lowest values were recorded under all severe drought treatments (I3), ranging from 36.35 to 49.43 mmol m^−2^ s^−1^ in I3P10 and I3P25 treatments, respectively.

#### 3.2.2 Chlorophyll index

**Figure 6:**
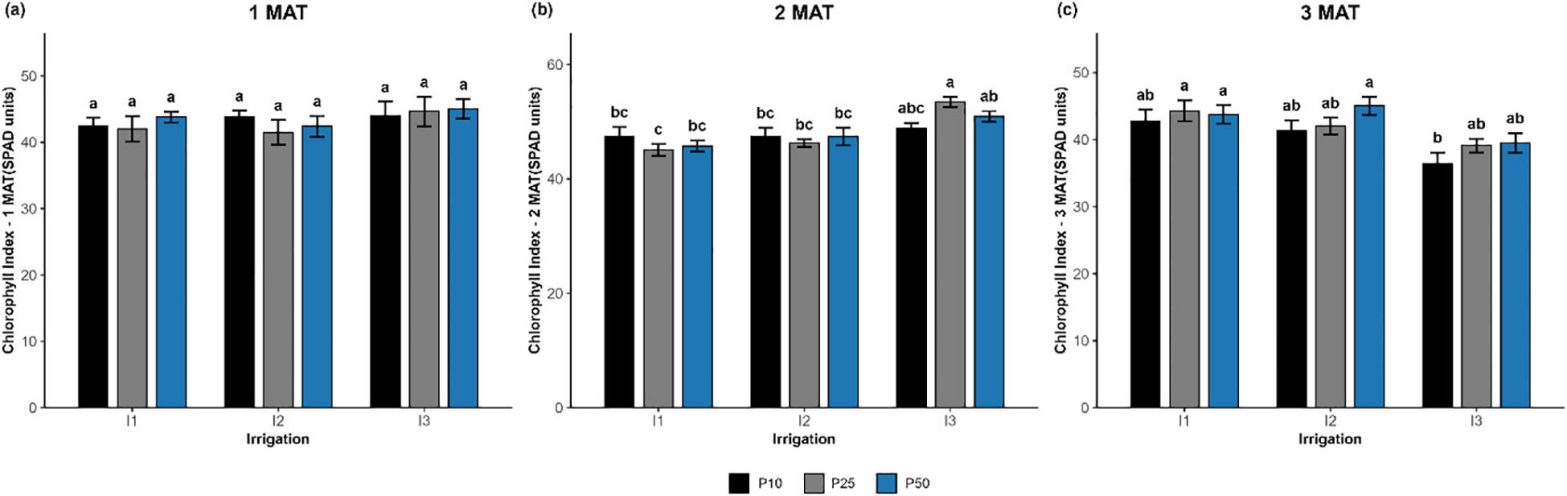
Evolution of the effect of different levels of drought stress and phosphorus doses on the chlorophyll index of *R. officinalis*: **(a)** 1MAT (month after transplantation), **(b)** 2 MAT, and **(c)** 3 MAT. bars represent mean values ± standard deviation. Bars sharing the same letter are not significantly different according to Tukey’s HSD test (P < 0.05).

In the 3^rd^ MAT, irrigation showed a significant effect *(p = 0.035)*, phosphorus showed a borderline effect *(p = 0.082*; **Figure 6**), and the interaction was not significant *(p = 0.710)*. The highest chlorophyll index was recorded in I2P50 (45.05 SPAD), I1P25 (44.31), and I1P50 (43.76), which were not significantly different. The lowest value was observed in I3P10 (36.39 SPAD). Equally, at 2^nd^ MAT, irrigation had a highly significant effect *(p < 0.001)*, and the interaction was also significant *(p = 0.033)*, while the phosphorus effect remained non-significant *(p = 0.915)*. On the other hand, at 1^st^ MAT, there were no significant effects of irrigation *(p = 0.698)*, phosphorus *(p = 0.714)*, or their interaction *(p = 0.842)* on the chlorophyll index.

#### 3.2.3 Chlorophyll *a*, *b*, and carotenoids

Chlorophyll *a* content (**Figure 3a**) in *R. officinalis* was significantly influenced by irrigation levels (p < 0.001), phosphorus fertilization (*p = 0.0013*), and their interaction (*p < 0.001*). Among the treatment combinations, I1P25 resulted in the highest chlorophyll concentration, climbing approximately to 9.93 mg/g Fresh Weight (FW), outperforming all other treatments. Treatments such as I1P50, I2P10, I1P10, I2P50, and I2P25 exhibited intermediates values between 4.75 and 6.13 mg/g FW, forming a homogeneous statistical group. Conversely, all treatments under drought stress (I3) exhibited markedly lower chlorophyll concentrations. In particular, I3P10 and I3P25 showed the lowest levels, decreasing to 1.6 and 0.9 mg/g FW, respectively, and were statistically distinct from all other treatments. Similarly, chlorophyll b content (**Figure 3b**) was significantly affected by irrigation (*p < 0.001*), phosphorus *(p = 0.0015)*, and their interaction *(p = 0.0163)*. The highest chlorophyll b level was recorded under treatment I1P25, achieving 4.67 mg/g FW, which was significantly greater than all other treatments and grouped separately. Treatments such as I2P50 and I2P25 exhibited comparable levels, measuring between 2.92 and 4.24 mg/g FW, while I1P10, I2P10, and I1P50 fell into intermediate statistical groups. The lowest pigment concentrations were observed under the I3 treatments, particularly I3P25, which had the lowest mean value of 0.45 mg/g FW. Although I3P50 and I3P10 showed marginally higher values, they did not differ significantly from the lowest group. Along the same lines, the carotenoid content (**Figure 3c**) was significantly influenced by irrigation level (*p < 0.001*) and their interaction (*p = 0.0034*), while phosphorus was not statistically significant (*p = 0.2018*). The treatment I1P25 maintained the highest carotenoid accumulation, grouped as the top performer. I2P50 followed closely, while a cluster of treatments including I1P50, I1P10, I2P10, and I2P25 ranged between 1.34 and 1.58 mg/g FW, while I3P50 occupied intermediate positions (0.86 mg/g FW). I3P10 and I3P25, both under limited irrigation (I3), exhibited the lowest carotenoid levels (0.68 and 0.37 mg/g FW), grouped together in the lower group. Overall, optimal phosphorus dose (P25) under well-watered condition (I1) and moderate drought (I2) resulted in the highest pigment *a* and *b* content.

#### 3.2.4 Phosphorus uptake

**Figure 7:**
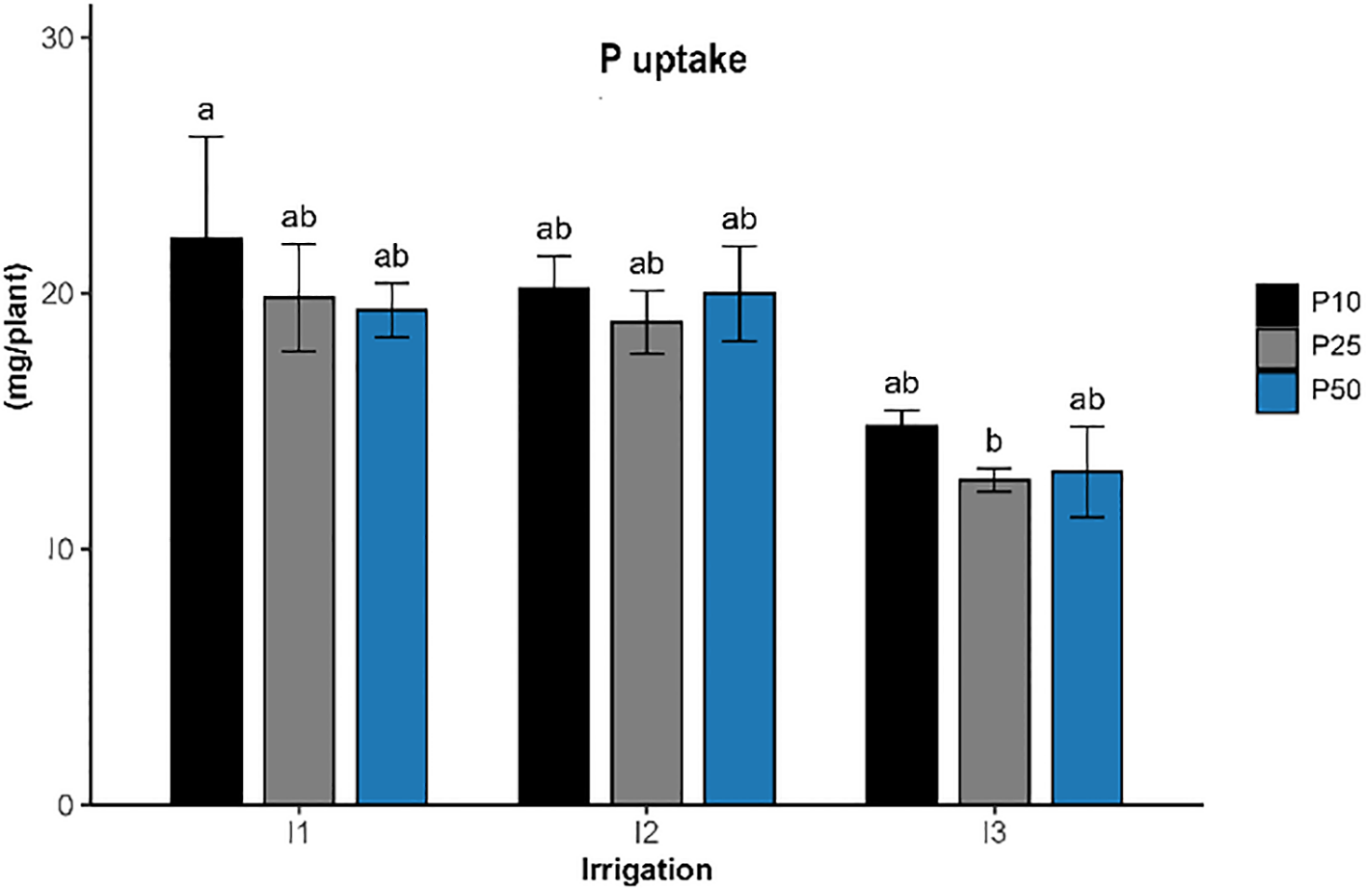
Effect of different levels of drought stress and phosphorus doses on P uptake of *R. officinalis*, bars represent mean values ± standard deviation. Bars sharing the same letter are not significantly different according to Tukey’s HSD test (*P < 0.05*).

Phosphorus uptake (**Figure 7**) in *R. officinalis* was significantly influenced by irrigation levels (p < 0.001), while phosphorus fertilization (p > 0.05) and their interaction (p > 0.05) showed no significant effects. The highest phosphorus uptake, reaching approximately 23.3 mg/plant, was recorded under well-watered conditions and low phosphorus level (1P10), and was significantly different compared to the treatments under severe drought stress and optimal phosphorus level (I3P25), which recorded the lowest uptake at around 16.2 mg/plant. Treatments including I1P25, I1P50, I2P10, I2P25, and I2P50 showed intermediate uptake values ranging from 19 to 21 mg/plant. All treatments under severe drought conditions (I3) generally resulted in reduced phosphorus uptake, while phosphorus application levels alone had limited influence under the tested conditions.

### 3.3 Biochemical traits

#### 3.3.1 Essential oil yield

**Figure 8:**
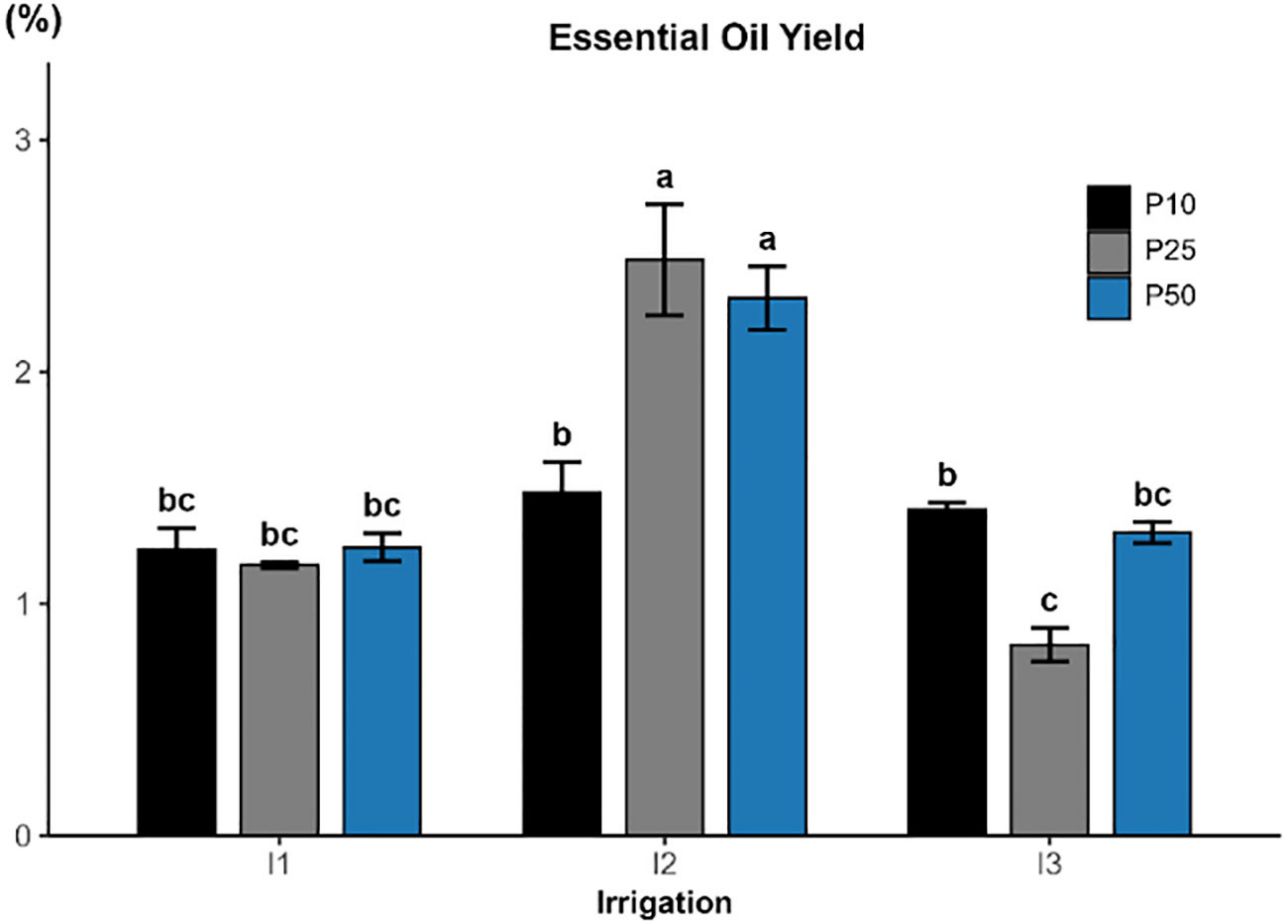
Effect of different levels of drought stress and phosphorus doses on essential oil yield in *R.officinalis*, bars represent mean values ± standard deviation. Bars sharing the same letter are not significantly different according to Tukey’s HSD test (*p* < 0.05).

Essential oil yield was significantly influenced by irrigation (p < 0.001; **Figure 8**), while phosphorus fertilization also had a significant but comparatively smaller effect (p = 0.0455). Additionally, the interaction was highly significant (p < 0.001), indicating that the effect of phosphorus varied depending on water availability. The highest yields were recorded under moderate drought conditions, achieving 2.48% under I2P25 and 2.32% under I2P50. In contrast, the lowest yield occurred under severe drought and medium P level (I3P25 = 0.82%), highlighting the negative effects of excessive water stress. However, treatments under full irrigation (I1) produced moderate yields between 1.16 and 1.24%, filling into an intermediate group.

#### 3.3.2 Essential oil composition

The result of essential oil composition in *R. officinalis* across different combinations of irrigation and phosphorus treatments exhibits a rich composition with a total of twenty-eight volatile compounds identified. These compounds varied significantly in response to irrigation, phosphorus availability, and their interaction (**Table 1**). Major constituents are α-pinene, β-pinene, eucalyptol, camphor, verbenone, D-limonene, endo-borneol, L-α-terpineol, bornyl acetate, and linalool, depending on the treatment. The other compounds, such as α-thujene, dehydrosabinene, o-cymene, terpineol, myrtenol, and others, were present in lower concentrations and thus classified as minor constituents.

**Table 1:** Essential oil compounds (%) in *R. officinalis* under different irrigation (I) and phosphorus treatments (P).

Compound	I1			I2			I3					
	P10	P25	P50	P10	P25	P50	P10	P25	P50	*P value (I)*	*P value(P)*	P value(I*P)
Tricyclene	0.18 ± 0.03a	0.13 ± 0.02b	0.15 ± 0.06ab	0.09 ± 0.05a	0.09 ± 0.02b	0.14 ± 0.01ab	0.15 ± 0.04a	0.16 ± 0.02b	0.12 ± 0.01ab	0.0148 *	0.6269	0.0958
Alpha_thujene	0.1 ± 0.02a	0.05 ± 0.01b	0.07 ± 0.03ab	0.04 ± 0.01a	0.05 ± 0.01b	0.06 ± 0.01ab	0.05 ± 0.01a	0.05 ± 0.01b	0.07 ± 0.02ab	0.00573 **	2.07427	0.02517 *
Alpha-Pinene	44.68 ± 4.57a	34.47 ± 0.41b	32.82 ± 4.42ab	27.57 ± 10.07a	23.48 ± 1.87b	35.69 ± 7.96ab	40.14 ± 9.02a	32.72 ± 2.23b	33.6 ± 1.79ab	0.0155 *	0.0498 *	2.0728
Camphene	5.28 ± 0.77a	3.94 ± 0.23b	4.35 ± 1.6a	3.25 ± 0.95a	3.01 ± 0.4b	4.3 ± 0.74a	4.89 ± 0.91a	5.26 ± 0.28b	4.28 ± 0.15a	0.00739 **	0.56827	0.07806
Dehydrosabinene	0.73 ± 0.11a	0.51 ± 0.06b	0.69 ± 0.29ab	0.44 ± 0.12a	0.48 ± 0.11b	0.55 ± 0.06ab	0.63 ± 0.11a	0.76 ± 0.05b	0.47 ± 0.03ab	0.0398 *	0.8956	0.0290 *
beta_Pinene	2.01 ± 0.1a	1.33 ± 0.11b	1.46 ± 0.71ab	1.01 ± 0.17a	0.86 ± 0.09b	1.19 ± 0.12ab	1.08 ± 0.08a	0.99 ± 0.17b	1.8 ± 0.09ab	0.000759***	0.008831**	0.009650**
beta _Myrcene	1.35 ± 0.1ab	0.95 ± 0.02b	0.99 ± 0.53a	0.84 ± 0.12ab	0.72 ± 0.08b	1.04 ± 0.1a	1.2 ± 0.31ab	1.45 ± 0.44b	1.25 ± 0.1a	0.00954 **	0.75999	0.15849
Thujene	0.32 ± 0.04ab	0.22 ± 0.02b	0.27 ± 0.11a	0.22 ± 0.03ab	0.19 ± 0.02b	0.24 ± 0.02a	0.29 ± 0.05ab	0.35 ± 0.11b	0.27 ± 0.02a	0.0183 *	0.7217	0.1477
Carene	0.93 ± 0.16ab	0.77 ± 0.02b	0.72 ± 0.21a	0.67 ± 0.13ab	0.66 ± 0.07b	0.85 ± 0.07a	0.96 ± 0.25ab	1.2 ± 0.39b	0.91 ± 0.05a	0.0106 *	0.8512	0.1548
o_Cymene	0.84 ± 0.1b	0.58 ± 0.02b	0.78 ± 0.42a	0.85 ± 0.37b	0.63 ± 0.11b	0.72 ± 0.08a	1.22 ± 0.43b	1.71 ± 0.5b	0.61 ± 0.04a	0.0058 **	2.1094	0.0103 *
D_Limonene	3.74 ± 0.54b	2.56 ± 0.1b	2.82 ± 0.62a	2.73 ± 0.43b	1.84 ± 0.71b	2.9 ± 0.55a	4.45 ± 0.42b	4.57 ± 1.84b	3.03 ± 0.23a	0.00185 **	0.11698	0.08238
Eucalyptol	22.82 ± 6.37a	25.22 ± 1.42a	24.25 ± 1.75a	22.05 ± 8.02a	25.31 ± 2.44a	25.95 ± 1.63a	22.78 ± 3.88a	22.08 ± 0.79a	25.61 ± 0.78a	0.872	0.345	0.828
gamma_Terpinene	1.89 ± 0.16a	1.33 ± 0.03a	1.43 ± 0.61a	1.27 ± 0.25a	1.1 ± 0.14a	1.31 ± 0.18a	1.25 ± 0.07a	1.64 ± 0.61a	1.65 ± 0.07a	0.0842	0.7016	0.1202
apha_Terpinolen	1.11 ± 0.12a	0.89 ± 0.02a	0.89 ± 0.4a	0.89 ± 0.25a	0.75 ± 0.09a	0.91 ± 0.09a	1.02 ± 0.27a	1.39 ± 0.62a	1.1 ± 0.05a	0.0729	0.9329	0.3651
Linalool	1.43 ± 0.35b	2 ± 0.07a	2.36 ± 0.36b	2.51 ± 0.5b	3.16 ± 0.37a	1.93 ± 0.58b	1.53 ± 0.82b	2.16 ± 0.38a	1.91 ± 0.21b	0.0100 *	0.0304 *	0.0443 *
Camphor	2.46 ± 0.23b	4.99 ± 0.24a	4.54 ± 1.71b	5.36 ± 1.12b	7.14 ± 0.36a	4.67 ± 1.76b	3.44 ± 1.73b	3.84 ± 1.21a	4.96 ± 0.32b	0.00797 **	0.03106 *	2.08366
Pinocarvone	0.39 ± 0.06b	0.66 ± 0.01a	0.63 ± 0.15b	0.71 ± 0.1b	0.96 ± 0.17a	0.6 ± 0.22b	0.44 ± 0.27b	0.55 ± 0.14a	0.68 ± 0.07b	0.0205 *	0.0335 *	2.0909
endo_Borneol	1.91 ± 0.26b	4.31 ± 0.44a	4.34 ± 2.08b	5.59 ± 1.64b	7.29 ± 0.87a	3.73 ± 1.78b	2.95 ± 1.38b	4.29 ± 2.01a	3.96 ± 0.28b	0.0116 *	0.0346 *	2.0945
Terpineol	0.63 ± 0.08b	1.15 ± 0.1a	1.29 ± 0.4b	1.38 ± 0.2b	1.78 ± 0.19a	0.99 ± 0.37b	0.83 ± 0.36b	1.1 ± 0.33a	1.22 ± 0.09b	0.0161 *	0.0178 *	0.0139 *
L_α _Terpineol	1.39 ± 0.35b	3.36 ± 0.46a	3.25 ± 1.59b	4.07 ± 0.89b	5.28 ± 0.47a	2.97 ± 1.45b	2.11 ± 0.93b	2.78 ± 1.33a	3.24 ± 0.3b	0.00854 **	0.04127 *	2.07464
Myrtenol	0.06 ± 0.02a	0.12 ± 0.04a	0.13 ± 0.05a	0.13 ± 0.08a	0.23 ± 0.05a	0.12 ± 0.09a	0.08 ± 0.04a	0.13 ± 0.07a	0.11 ± 0.04a	2.082	0.0478 *	2.4642
Borneol	0.22 ± 0.1b	0.49 ± 0.1a	0.46 ± 0.21ab	0.73 ± 0.46b	0.87 ± 0.16a	0.44 ± 0.24ab	0.3 ± 0.15b	0.51 ± 0.26a	0.45 ± 0.08ab	0.0257 *	0.134	0.2798
Verbenone	2.04 ± 0.49b	5.91 ± 0.57a	5.24 ± 2.38b	5.73 ± 1.09b	7.13 ± 0.61a	4.9 ± 1.69b	3.2 ± 1.49b	2.74 ± 0.83a	5.35 ± 0.28b	0.00474 **	0.02118 *	0.00677 **
Citronellol	0.13 ± 0.03a	0.2 ± 0.07a	0.23 ± 0.06a	0.51 ± 0.51a	0.4 ± 0.09a	0.2 ± 0.1a	0.16 ± 0.08a	0.37 ± 0.09a	0.21 ± 0.05a	0.134	0.463	0.281
Geraniol	0.45 ± 0.24a	0.95 ± 0.21a	0.88 ± 0.42a	1.39 ± 0.87a	1.52 ± 0.36a	0.78 ± 0.48a	0.51 ± 0.34a	0.97 ± 0.44a	0.88 ± 0.14a	0.0612	0.1992	0.2778
Bornyl acetate	1.45 ± 0.27a	1.61 ± 0.07a	2.4 ± 0.71a	4.38 ± 5.21a	2.5 ± 0.24a	1.51 ± 0.1a	2.22 ± 0.48a	2.83 ± 0.27a	1.44 ± 0.1a	0.508	0.566	0.395
Thymol	0.16 ± 0.12b	0.2 ± 0.11a	0.18 ± 0.09ab	0.91 ± 1.12b	0.78 ± 0.33a	0.53 ± 0.42ab	0.4 ± 0.19b	0.95 ± 0.19a	0.04 ± 0.02ab	0.0405 *	0.1765	0.398
Caryophyllene	1.29 ± 0.43a	1.11 ± 0.3a	2.37 ± 0.99a	4.67 ± 6.54a	1.79 ± 0.3a	0.82 ± 0.14a	1.73 ± 0.22a	2.47 ± 0.64a	0.76 ± 0.07a	0.681	0.501	0.337

Data represents means ± SD. different letters mean significant difference according to Tukey’s HSD test (*p <0.05).*

I1: Well-watered; I2: Moderate drought stress; I3: Severe drought stress; P10: 10 kg/ha P; P25: 25 kg/ha P; P50: 50 kg/ha P.

The symbol “*” in **Table 1** indicates that the corresponding mean values are significantly different according to the statistical test.

Drought stress significantly influenced 20 of the identified compounds out of 28 evaluated (p < 0.05), particularly affecting both major and minor constituents. Mainly, compounds such as camphene, β-myrcene, and D-limonene exhibited highly significant responses *(p < 0.01)*, emphasizing the strong effect of drought on monoterpene accumulation. Phosphorus fertilization had significantly influenced 10 compounds.

**Figure 9:**
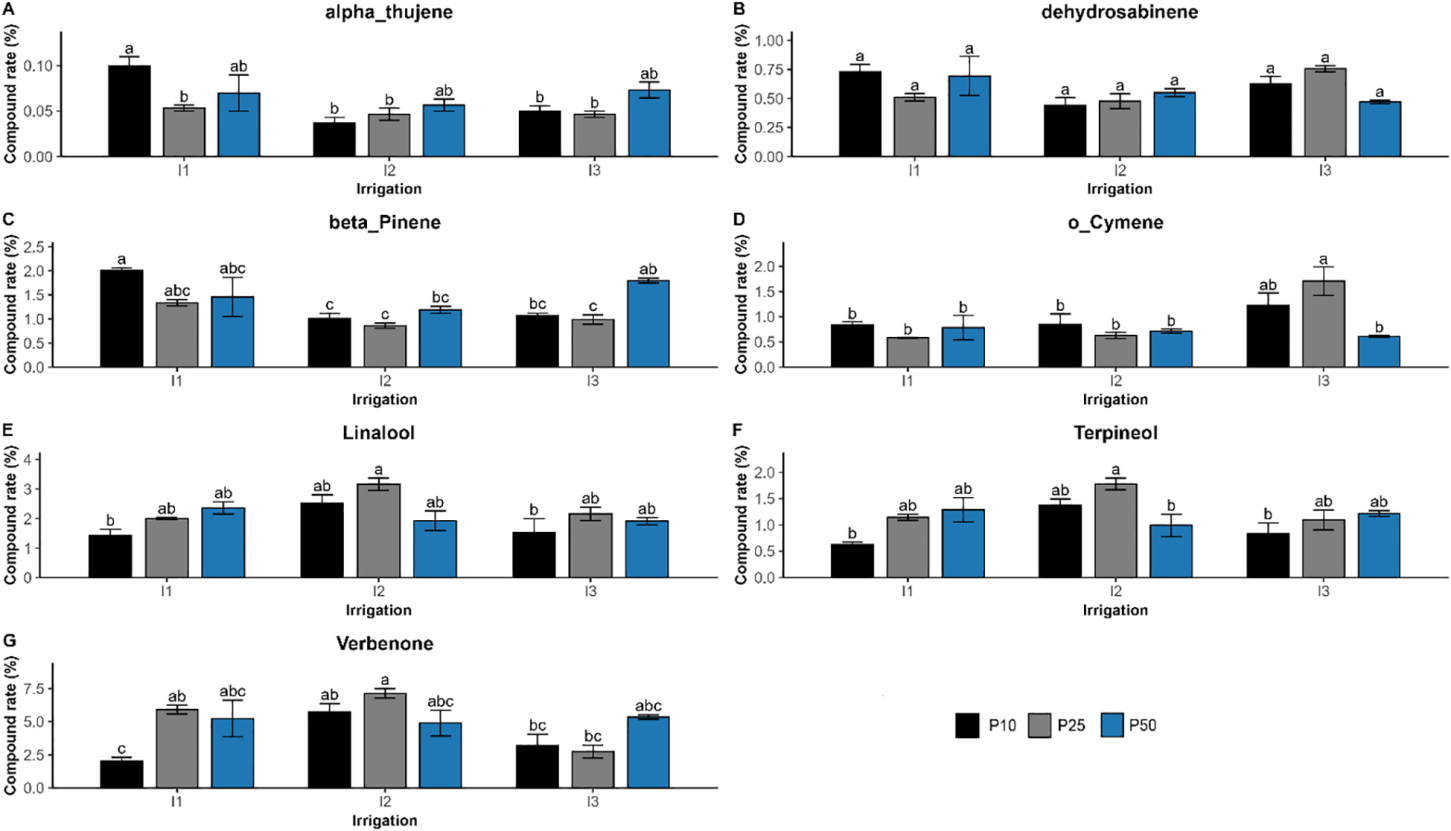
Effect of different levels of drought stress and phosphorus doses on essential oil compounds in *R. officinalis*: **(A)** alpha-Thujene, **(B)** Dehydrosabinene, **(C)** beta_Pinene, **(D)** o_Cymene, **(E)** Linalool, **(F)** Terpineol, and **(G)** Verbenone. bars represent mean values ± standard deviation. Bars sharing the same letter are not significantly different according to Tukey’s HSD test (*p* < 0.05).

**Figure 10:**
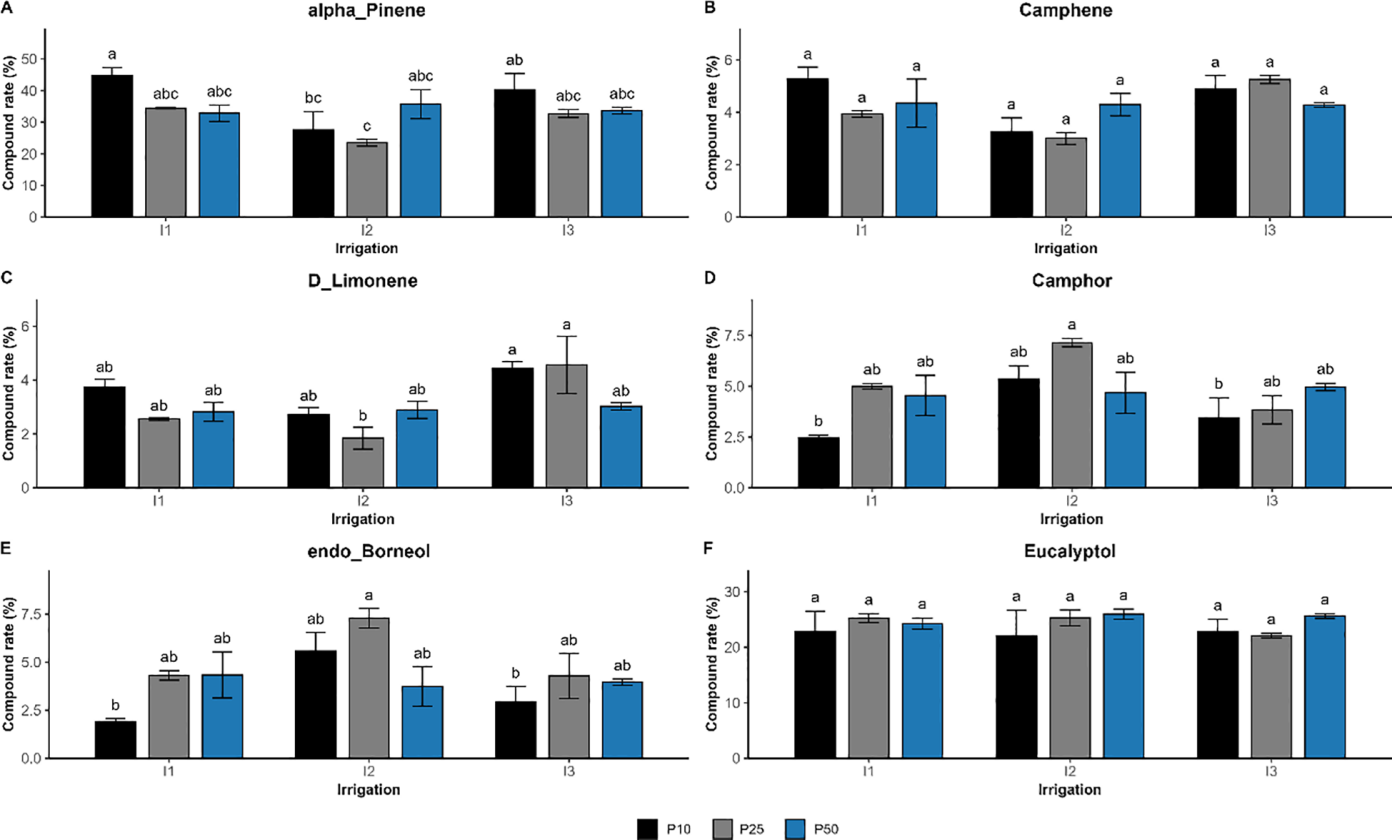
Effect of different levels of drought stress and phosphorus doses on major essential oil compounds in *R. officinalis*: **(A)** alpha-Pinene, **(B)** Camphene, **(C)** D_Limonene, **(D)** Camphor, **(E)** Endo_Borneol, and **(F)** Eucalyptol. bars represent mean values ± standard deviation. Bars sharing the same letter are not significantly different according to Tukey’s HSD test (*p* < 0.05).

The interaction of drought stress and phosphorus fertilization significantly influenced the composition of seven compounds *(p < 0.01)*, such as verbenone, β-pinene, linalool, o-cymene, dehydrosabinene, terpineol, and α-thujene (**Figure 9**). Their concentration reflected a synergistic regulation, showing that the effect of one factor was significantly dependent on the level of the other. Verbenone concentration peaked at 7.13% under moderate drought and optimal phosphorus dose (I2P25), which is much higher than well-watered (I1P10; 2.04%) and severe drought conditions. Similar trends were seen for linalool and terpineol, which also peaked at I2P25 at concentrations of 3.16% and 1.78%, respectively. However, β-pinene showed a distinct pattern. It peaked in well-watered conditions with low phosphorus levels (I1P10, 2.01%), dropped in drought-stressed conditions (I3P10, 1.08%), and remained relatively high under I3P50. Other major compounds were significantly influenced by irrigation or phosphorus or both, but not their interactions (**Figure 10**). Specifically, camphor, l-α-terpineol, and endo-borneol were strongly affected by both irrigation and phosphorus levels, reaching 7.14%, 5.28%, and 7.29%, respectively, and were highest under moderate drought and optimal phosphorus doses (I2P25). In contrast, α-pinene and camphene peaked under well-watered and low-phosphorus levels (I1P10), attaining 44.68% and 5.28%, respectively. On the other hand, D-limonene and o-cymene concentrations increased under severe drought and optimal phosphorus level (I3P25), climbing to 4.57% and 1.71%, respectively. Eucalyptol showed no significant changes in response to either irrigation or phosphorus levels, nor their interaction *(p > 0.3)*.

**Figure 11:**
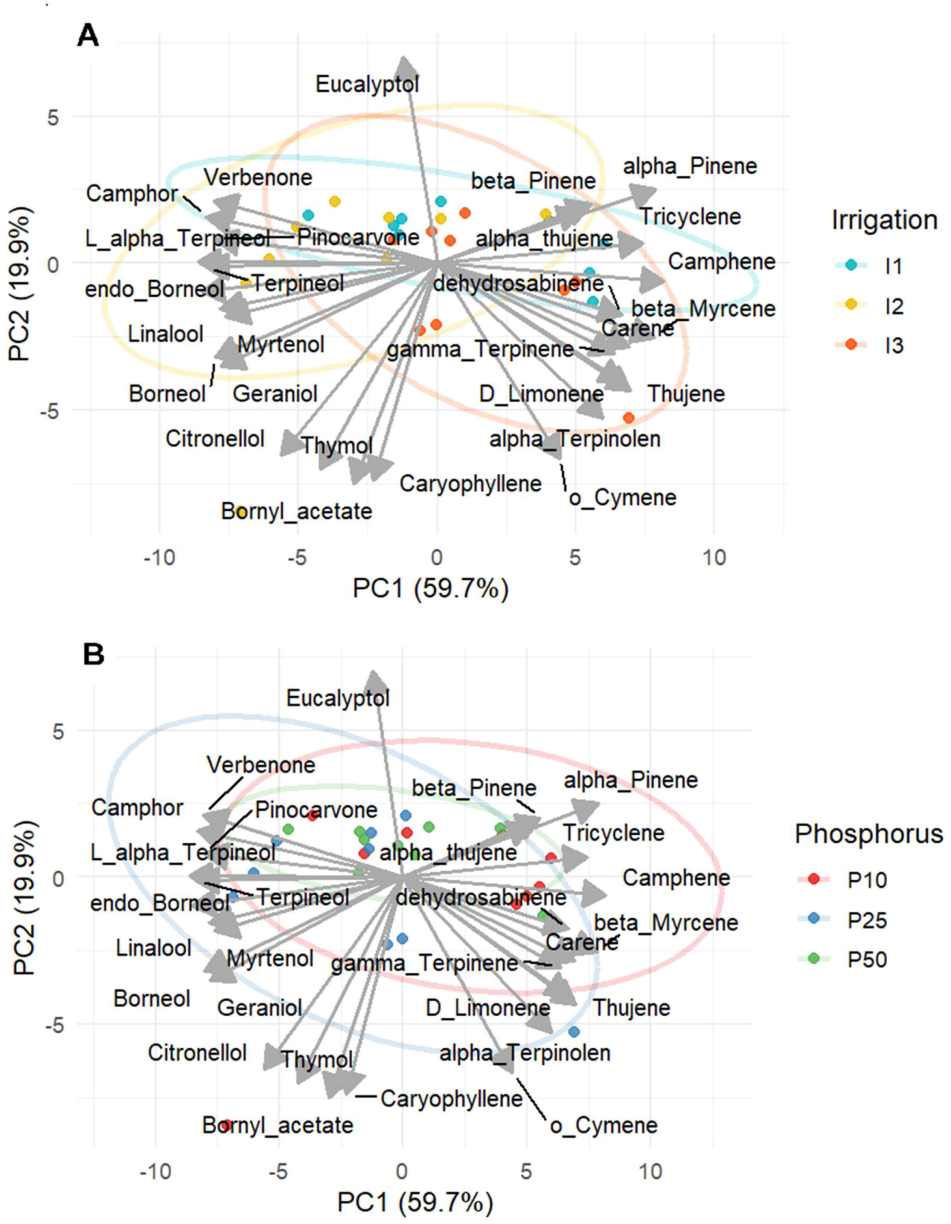
PCA of Essential Oil Composition of *R. officinalis* under different irrigation **(A)** and phosphorus treatments **(B)**.

A principal component analysis (PCA) was conducted to better understand the behavior of different variables evaluated and to assess the treatment impact on essential oil profiles based on 28 compounds funded. The first two principal components (PC1 and PC2) accounted for 59.7% and 19.9% of the total variance, respectively, capturing a cumulative 79.6% of the variation in EO profiles. The PCA based on irrigation levels (**Figure 11A**) revealed a clearly different treatment cluster. Under well-watered conditions (I1), samples were closely associated with non-oxygenated monoterpenes such as α-pinene, β-pinene, tricyclene, and α-thujene. Under moderate drought stress samples (I2) were aligned with oxygenated compounds such as endo-borneol, verbenone, borneol, linalool, and camphor consistent with ANOVA results. On the other hand, under severe drought (I3) samples were arranged next to D-limonene, o-cymene, α-terpinolen, and γ-terpinene. Moreover, compounds such as eucalyptol were plotted orthogonally to the main irrigation-based separation. Phosphorus based PCA (**Figure 11B**) showed that moderate phosphorus (P25) clustered near verbenone, linalool, and camphor, endo-borneol consistent with ANOVA results highlighting their optimal synthesis at balanced phosphorus levels. Conversely low phosphorus (P10) was associated with compounds such as α-pinene, camphene, β-myrcene. Whereas high phosphorus level (P50) showed an overlap with unclear separation between treatment. These PCA findings reveal that phosphorous serves as a secondary modulator, under stress combinate condition, and the irrigation is the clear driver forming essential oil profiles. Overall, both PCA analyses on irrigation and phosphorus indicate that a metabolic balance is reached under moderate irrigation (I2) and phosphorus fertilization (P25), which improves biosynthesis of oxygenated monoterpenes in rosemary.

## 4 Discussions

### 4.1 Water availability overrides phosphorus in shaping morphological and root growth in rosemary

The study revealed that irrigation levels significantly affected all morphological traits, including leaf area index, stem diameter and length, shoot and root dry biomass, and root traits. In contrast, phosphorus application significantly influenced LAI and stem diameter (**Figure 1**), especially under well-watered (I1) and moderate drought (I2), but under extreme drought circumstances (I3), this effect diminished, revealing a more limited and context-dependent effect. This pattern can be attributed to water deficit in limiting leaf expansion by lowering turgor pressure and reducing carbon assimilation, thereby overriding the potential benefits of phosphorus on growth (Farooq et al., 2009; Raffo et al., 2020). Both irrigation and phosphorus clearly influenced stem diameter during early phases of development. Plants kept under well-watered conditions consistently had the thickest stems. In parallel, phosphorous application greatly expanded diameter at 1^st^ and 3^rd^ MAT. This response indicates that phosphorus plays a key role in the first stage of shoot and root establishment (**Figure 1**), potentially through its involvement in energy metabolism (ATP), nucleic acid synthesis, and membrane integrity (Gutiérrez-Boem and Thomas, 2001). In contrast, excessive phosphorus fertilizer (P50) during drought may adversely impacted biomass by inhibiting photosynthetic efficiency (Vukmirović et al., 2024).

Stem length and shoot dry biomass were significantly influenced by drought stress. Shoot dry biomass remained relatively stable under well-watered and moderate drought conditions, reflecting the cumulative advantages of sufficient water availability. This may demonstrate that in the early phase of development of rosemary, moderate drought has minimal effect on biomass accumulation (Formica et al., 2024). Conversely, under drought stress, significant declines in stem length and shoot biomass were recorded due to lowered water availability and turgor pressure (Bidgoli, 2018; Ghamarnia et al., 2022).

Root dry biomass significantly increased under moderate drought conditions (**Figure 2**) across all I2 treatments. These results indicate an adaptive focus on root development to enhance water absorption in conditions of limited water availability (Abbaszadeh et al., 2020; Bidgoli, 2018). In contrast, severe drought (I3P10) and (I3P50) resulted in decreased root biomass, signifying a restricted ability to sustain development under high stress. The lack of a notable phosphorus effect indicates that water availability was the primary controlling factor affecting root development. The modest positive correlation between root and shoot biomass (*r = 0.275, p = 0.0047*) indicates a largely coordinated development pattern, suggesting a tendency for root prioritization under drought conditions, aligning with established adaptive techniques (Smirnoff, 1998). Similarly, the same pattern was observed in *Medicago sativa*, where drought stress diminished biomass but stimulated root elongation to cope with it (Farooq et al., 2009). Root length and root surface area exhibited a comparable trend (**Figure 4**), demonstrating a considerable increase under severe drought conditions (I3), with the most notable elongation observed in the I3P50 treatment, supporting water uptake. In contrast, the average root diameter exhibits a distinct decline under drought conditions, with the most substantial roots seen in I1P50, highlighting water availability, and the thinnest roots in I3P50. This result aligns with the finding of Yang et al. (2021), who observed that drought stress may impede the development of cotton seedlings while promoting the elongation and thickness of fine roots.

### 4.2 Physiological adaptations of *R. officinalis* to drought and phosphorus availability

Water availability and phosphorus significantly affected *R. officinalis* physiological traits, including stomatal conductance, chlorophyll index, chlorophyll *a*, chlorophyll *b, and* carotenoids. In contrast, P uptake was exclusively influenced by irrigation. The results confirmed that phosphorus played a more variable role, usually as a secondary function. Both phosphorus and severe drought clearly influenced stomatal conductance (**Figure 5**). Although no significant interaction between treatments was observed, the result suggests that both probably influence stomatal behavior independently. Under drought stress, rosemary showed significantly reduced stomatal conductance and photosynthetic efficiency, regardless of nutrient availability. This decline in gas exchange capacity and photosynthetic efficiency is consistent with stress-induced stomatal closure and decreased photosynthesis, ultimately limiting growth potential (Nogués et al., 2015; Chaves et al., 2003). Optimal phosphorus (P25) under moderate drought (I2) helped maintain a relatively high level of gas exchange, nearly comparable to well-watered plants. This suggests that phosphorus may help to regulate rosemary physiology, preserving stomatal conductance and lowering abscisic acid (ABA) buildup, hence improving water-use efficiency in plants under abiotic stress (Khan et al., 2023).

By the 3^rd^ MAT, the chlorophyll index results revealed a decrease in chlorophyll accumulation under severe drought stress conditions (**Figure 6**). This can be attributed to the degradation of photosynthetic pigments and chloroplast cell death, confirming that severe drought greatly compromises pigment stability and cellular integrity. The drop in oxidative degradation serves as a physiological stress adaptation mechanism (Jamaladdeen et al., 2023; Munné-Bosch et al., 2002). The highest pigment concentrations were consistently recorded under well-watered conditions and optimal phosphorus doses (I1P25), suggesting that well-watered conditions (I1) together with moderate phosphorus supplies (P25) maximize pigment biosynthesis (**Figure 3**). Remarkably, both chlorophyll *a* and *b* levels remained relatively high under moderate drought and moderate phosphorus levels (I2P25), suggesting that moderate phosphorus doses (P25) help maintain high pigment accumulation under well-watered and moderate drought conditions. In contrast, phosphorus deficiency lowers ATP availability and slows PSII electron transport, processes most likely to contribute to lower chlorophyll concentration under inadequate phosphorus availability (Jacob and Lawlor, 1993). This finding is consistent with the study of Warren (2011), who showed that P deficiency can limit metabolic activities, including Rubisco carboxylation capacity, thereby impairing photosynthetic efficiency. Furthermore, the study of Sharawy Mostafa (2019) demonstrated that chlorophyll pigments increased under moderate organic and inorganic inputs, highlighting the beneficial effect of a balanced nutrient supply combined with moderate water conditions. In contrast, severe drought stress (I3) combined with either low, optimal, or high phosphorus doses exhibited a significant decline in all pigments, highlighting the dominant negative impact of water constraint on pigment accumulation.

Phosphorus uptake (**Figure 7**) in *R. officinalis* was significantly influenced by irrigation levels. Both treatments under well-watered and moderate conditions maintained high P uptake levels, indicating that moderate stress did not impair the plant’s ability to acquire phosphorus. This reflects its high nutrient acquisition capacity due to greater biomass and a more active root system (Lynch, 2011). However, P uptake was significantly decreased across treatments under drought stress. On the other hand, raising phosphorus levels did not considerably increase phosphorus uptake, especially under drought stress conditions. This suggests that the phosphorus supply was insufficient to overcome the physiological constraints imposed by water deficit. These are aligned with earlier findings that demonstrated drought inhibits plant nutrient uptake pathways (Bista et al., 2018).

### 4.3 Complex interactions between phosphorus and drought stress regulate essential oil composition in *R. officinalis*

Essential oil analysis in *R. officinalis* under varying irrigation and phosphorus treatments revealed a total of 28 chemical compounds, with distinct shifts in composition depending on stress level and phosphorus availability. The combined effect of moderate drought stress (I2) and optimal phosphorus supplementation significantly enhanced both yield and composition of essential oil (**Figure 7**; **Table 1**). The result on essential oil yield of this study clearly demonstrates that irrigation is the most influential factor affecting essential oil yield in *R. officinalis*. The highest oil yield was obtained under moderate drought stress (I2), particularly when combined with optimal phosphorus doses (P25). Essential oil yield increased by up to 113%, reaching 2.48% compared to the well-watered conditions (I1P25), highlighting a combined effect of balanced stress and nutrient supply. While the lowest yield occurred under severe drought (I3P25). These results emphasize that moderate water stress, when combined with adequate phosphorus, can stimulate secondary metabolism and terpenoid biosynthesis without compromising biomass. This outcome is consistent with observed responses in related studies on rosemary under similar agronomic conditions (Bidgoli, 2018; Formica et al., 2024; Sharawy Mostafa, 2019).

Our findings revealed that key monoterpenes, including endo-borneol, verbenone, linalool, l-α-terpineol, terpineol, camphor, α-pinene, and β-pinene, were significantly modulated by water availability and phosphorus levels. Peak concentrations of oxygenated monoterpenes such as endo-borneol, verbenone, linalool, l-α-terpineol, terpineol, and camphor were consistently recorded under moderate drought combined with moderate phosphorus (I2P25), indicating that their biosynthesis is favored under balanced stress and nutrient conditions. PCA confirmed this trend, clustering these compounds under I2 and P25 treatments (**Figures 10A, B**). This suggests that moderate drought coupled with optimal phosphorus supply promotes a shift in metabolic allocation toward the production of oxygenated monoterpenes, likely via oxidative signaling pathways. Endo-borneol attained 7.29% with I2P25, whereas compounds such as verbenone climbed to 7.13%. Linalool peaked at 3.16% under the same treatment, camphor was enhanced to 7.14%, terpineol exhibited an accumulation of 1.78%, and l-α-terpineol reached 5.28%, respectively. Drought stress is known to induce reactive oxygen species (ROS), which act as signaling molecules to enhance the expression of terpene synthase (TPS) genes, thereby increasing carbon flux into monoterpene biosynthesis and facilitating the accumulation of oxygenated volatiles like camphor (Radwan et al., 2017; Ramezani et al., 2020). The result is in agreement with Sarmoum et al. (2019) and Jamaladdeen et al. (2023) on rosemary. This also aligned with finding of Peçanha et al. (2021), who demonstrated that linalool accumulation is positively correlated with increased phosphorus fertilization in Lavandula angustifolia.

In contrast, α-pinene and β-pinene were most abundant under well-watered conditions (I1), particularly at low phosphorus (P10), reaching 44.68% and 2.01%, respectively. These non-oxygenated monoterpenes were clustered under I1 and P10 in PCA, indicating their association with baseline metabolism and suggesting that phosphorus limitation favors the production of primary volatiles, highlighting a positive relationship between non-oxygenated monoterpenes and photosynthesis (Nogués et al., 2015). Under severe drought and high phosphorus conditions (I3P50), β-pinene concentrations remained relatively high, suggesting that there may be a compensatory upregulation of biosynthesis during prolonged stress, possibly due to enhanced availability of phosphorus precursors. Meanwhile, D-limonene and o-cymene were predominantly influenced by irrigation, with both compounds increasing significantly under I3 treatments. D-limonene appeared to be specifically drought-inducible, potentially functioning in abiotic stress defense pathways (Boncan et al., 2020; Kulak, 2020). o-cymene accumulation was influenced by both water and phosphorus, peaking under I3P25, which points to its role in adaptive responses to drought. This is consistent with the study of Chrysargyris et al. (2016), who reported an increase in *o*-cymene concentrations under optimal phosphorus levels in *Lavandula angustifolia*.

Under severe drought, PCA positioned compounds like D-limonene, o-cymene, α-terpinolen, and γ-terpinene toward the left and lower quadrants, suggesting their involvement in stress-related metabolic pathways. Conversely, high phosphorus (P50) treatments produced neutral clustering near the origin, indicating a dampening of specific secondary metabolite responses and reinforcing the idea that excessive phosphorus may suppress adaptive secondary metabolism. Eucalyptol levels remained relatively stable across treatments, showing minimal sensitivity to irrigation or phosphorus inputs. This stability was also evident in PCA plots, where eucalyptol was positioned orthogonally to irrigation-based separation, implying limited plasticity in response to environmental variation (Sarmoum et al., 2019).

## 5 Conclusion

The findings of this study demonstrated that drought stress significantly influenced the morphological traits, physiological responses, and essential oil yield and composition in *R.* officinalis, while phosphorus exerts a more limited and context-dependent effect. Under severe drought stress, morphological parameters such as leaf area index, stem diameter, stem length, shoot dry biomass, and root average diameter declined, indicating the sensitivity of rosemary to water stress, altering physiological traits such as stomatal conductance and chlorophyll content. However, shoot dry biomass remained relatively stable under moderate drought, and root traits exhibited some degree of compensatory adaptation, likely to support water uptake. Phosphorus supplementation, though beneficial under well-watered or moderate drought scenarios, had a comparatively secondary role, showing minimal effects under severe drought. Physiologically, moderate phosphorus supply (P25) under moderate water stress (I2) helped maintain stomatal conductance and chlorophyll content, promoting resilience in photosynthetic processes. Phosphorus and drought stress had an impact on both yield and essential oil composition without compromising biomass. Moderate drought (I2) and optimal phosphorus (P25) recorded the highest yield (2.48%), which is 113% more than well-watered conditions (I1P25), and the highest accumulation of key oxygenated monoterpenes such as endo-borneol, verbenone, linalool, terpineol, and camphor. These compounds appear to reflect a metabolic shift toward enhanced secondary metabolite production under balanced stress. Conversely, nonoxygenated monoterpenes such as α-pinene and β-pinene were more abundant under well-watered conditions and low phosphorus levels, highlighting their association with baseline metabolism. In contrast, stress-inducible volatiles like D-limonene and o-cymene increased under severe drought, pointing to their potential roles in long-term stress adaptation and signaling. Overall, this research underscores the importance of moderate water stress and balanced phosphorus nutrition in optimizing both growth and biochemical responses in rosemary. These insights are particularly valuable for sustainable cultivation strategies in arid and semi-arid regions, where water management and adequate nutrient input can significantly influence plant performance and essential oil quality.

## Data Availability

The raw data supporting the conclusions of this article will be made available by the authors, without undue reservation.
